# Effects of β-Mannanase Supplementation and Soyhull Inclusion on Production Performance, Economics, Egg Quality, Blood Biochemicals, Nutrient Digestibility, and Intestinal Morphology in Golden Brown Hens (RIR × Fayoumi) during Late Peak Production

**DOI:** 10.3390/ani14142047

**Published:** 2024-07-12

**Authors:** Muhammad Shuaib, Abdul Hafeez, Muhammad Tahir, Abubakar Sufyan, Obaid Ullah, Muhammad Adnan Shams, Shahrood Ahmed Siddiqui, Ayman A. Swelum

**Affiliations:** 1 College of Animal Science and Technology, Yangzhou University, Yangzhou 225009, China; 2 Department of Poultry Science, Faculty of Animal Husbandry & Veterinary Sciences, The University of Agriculture, Peshawar 25000, Pakistan; hafeezpak@hotmail.com (A.H.); drmuhammadadnanshams@gmail.com (M.A.S.); 3 Department of Animal Nutrition, Faculty of Animal Husbandry & Veterinary Sciences, The University of Agriculture, Peshawar 25000, Pakistan; tahir065@yahoo.co.uk; 4 Department of Livestock and Poultry Production, Bahauddin Zakariya University, Multan 60800, Pakistan; linksufyan@bzu.edu.pk; 5 Department of Veterinary Clinical Sciences, Faculty of Veterinary and Animal Sciences, University of Poonch, Rawalkot 12350, Pakistan; obaid.vet@upr.edu.pk; 6 Vaccine Production Unit Sindh Tandojam, Livestock and Fisheries Department Government of Sindh, Karachi 70050, Pakistan; drshahroodsiddiqui@yahoo.com; 7 Department of Animal Production, College of Food and Agriculture Sciences, King Saud University, Riyadh 11451, Saudi Arabia

**Keywords:** feed intake, feces consistency, viscosity, FCR, Haugh unit

## Abstract

**Simple Summary:**

This study explored the effects of the different dietary combinations of soyhulls and the β-mannanase enzyme on production performance, economics, egg quality, blood biochemicals, nutrient digestibility, and gut health in laying hens during the late peak production phase. Golden brown hens were fed different diets for four weeks, and the combination of 3% soyhulls and 30 mg/kg β-mannanase showed potential benefits. It improved production performance and increased egg weight, albumen weight, and height while significantly lowering total cholesterol, LDL, and VLDL levels. This combination also improved gut morphology and enhanced nutrient digestibility. Overall, the inclusion of 3% soyhulls and 30 mg/kg β-mannanase in the diet may have positive effects on production performance, nutrient digestibility, and gut health and potentially lower serum cholesterol levels in laying hens while maintaining acceptable egg quality.

**Abstract:**

This study investigated the effects of the β-mannanase enzyme and soyhulls on production performance, economics, egg quality, hematology and serum biochemistry, nutrient digestibility, gut morphology, digesta viscosity, and excreta consistency in laying hens during the late peak production phase (37 to 40 weeks of age). Golden brown hens (RIR × Fayoumi; *n* = 200) were fed a control diet (no soyhulls or enzymes) and diets containing four combinations, i.e., 3% soyhulls with 20 mg/kg β-mannanase (D1), 3% soyhulls with 30 mg/kg β-mannanase (D2), 9% soyhulls with 20 mg/kg β-mannanase (D3), and 9% soyhulls with 30 mg/kg β-mannanase (D4), for four weeks in four replicates of 10 birds each. Overall, a significantly higher (*p* < 0.05) feed intake, weight gain, feed conversion ratio, and water intake were calculated in the D2 group as compared to the control and remaining combinations of soyhulls and β-mannanase. No mortality was recorded during the entire experiment. Economically, the D1 and D2 groups showed the best results as compared to the D3 and D4 groups. Egg quality parameters like egg weight, shell weight and shell thickness, yolk weight, albumen weight and height, and the Haugh unit remained unchanged (*p* > 0.05). Similarly, the D2 group showed significantly lower total cholesterol, LDL, and VLDL levels and enhanced gut morphology with greater villus width, height, crypt depth, and surface area across intestinal segments. Crude protein (CP), crude fiber (CF), crude fat, and ash digestibility were higher (*p* < 0.05) in the D1 and D2 groups compared to the control. Digesta viscosity, excreta consistency, and other egg quality parameters remained unaffected. In conclusion, the dietary inclusion of a combination of 3% soyhulls and 30 mg/kg β-mannanase may have potential benefits for laying hens by improving some production performance and egg quality indicators and economics, lowering blood cholesterol, LDL, and VLDL levels, enhancing nutrient digestibility, and improving gut morphology without affecting egg quality.

## 1. Introduction

The poultry industry faces a dual challenge: feeding the growing global population with protein while minimizing its environmental impact and production costs. A sustainable and cost-effective poultry production approach involves adopting practices that promote long-term ecological balance, resource conservation, and financial efficiency [1]. Formulating diets that cover the nutrient requirements of animals, including alternative feed ingredients such as soyhulls and corn distillers grains, presents ideas for producing animal-based food for human use in an efficient manner, with a substantial decrease in emissions of greenhouse gases [2]. In this context, dietary fiber represents a paradigm shift in sustainable poultry production. However, dietary fiber plays a complex role in the health and performance of poultry [3]. While insoluble fibers like cellulose and lignin are generally considered safe, their high levels can increase digesta retention time, potentially affecting nutrient absorption [4]. Soluble fibers like pectin and certain hemicelluloses, although beneficial for gut health, can lead to enteric problems at higher levels due to increased digesta viscosity [5].

Soyhulls, a by-product of oil extraction, are rich in insoluble fibers like lignocellulose but also contain varying levels of other fiber fractions [6]. Their rapid fermentation and the presence of beneficial sugars like galactomannan offer potential nutritional benefits [7,8,9]. However, soyhulls also contain high amounts of non-starch polysaccharides (NSPs), which can increase digesta viscosity, reduce the digestibility of nutrients, and depress growth performance [10]. Among NSPs, β-mannans are a group of related heat-resistant compounds that survive the drying–toasting phase of processing soybeans and make up 1.3 to 1.6% of dehulled or non-dehulled soybeans [11,12,13]. β-Mannans are mainly found in the hull and fiber fractions of soybean and are highly viscous and anti-nutritional [14]. Due to the lack of enzymes needed to efficiently digest the NSPs present in soyhulls, their utilization is limited in poultry [15]. Supplementation with exogenous enzymes like β-mannanase can address this challenge. These enzymes break down NSPs, reducing digesta viscosity and improving nutrient digestibility [16]. Therefore, β-mannanase can enhance the performance and production efficiency of poultry, making soyhulls more viable and cost-effective feed ingredients.

In the crucial peak production phase of laying hens, utilizing soyhulls and β-mannanase together in their diets holds the potential for both economic and performance benefits. Soyhulls, a cost-effective by-product of soybean processing, can partially replace soybean meal, lowering feed costs but also introducing indigestible fibers, and β-mannanase can break down β-mannans, improving nutrient digestibility and potentially increasing egg production without sacrificing egg quality. However, finding the optimal balance of soyhulls and β-mannanase requires further research, as higher soyhull levels might require more enzymes for optimal outcomes. This intriguing strategy could pave the way for sustainable and cost-effective laying-hen feed, increasing the farm profit while maintaining or even enhancing egg production during the peak period. It is therefore assumed that the addition of the enzyme β-mannanase (Hemicell^TD^) to a soybean-hull-based diet may break down β-mannans, compensate for the negative effect of the high levels of fiber in the soyhulls, and improve the nutrient digestibility without effecting the egg quality. This study aims to investigate the effects of different combinations of soyhulls and β-mannanase on various aspects of laying hens’ health and productivity during late peak egg production (33–36 weeks). By exploring the synergy between enzymes and soyhulls, this study seeks to contribute to the development of more sustainable and cost-effective feeding strategies for laying hens while ensuring optimal health and productivity.

## 2. Materials and Methods

### 2.1. Birds Housing, Experimental Diet, and Environment

A total of 200 golden brown (RIR × Fayoumi) laying hens at 36 weeks of age were purchased from a commercial market and weighed individually. The birds were randomly allocated to five groups of 40 birds each, with four replicates of 10 birds per group (five cages per replicate with 2 birds per cage). The experimental diets consisted of a control diet (no soyhulls or enzymes) and four treatment diets containing combinations of 3% or 9% soyhulls with 20 or 30 mg/kg β-mannanase (Hemicell™, Elanco Animal Health, Greenfield, IN, USA) (Table 1). All of the birds were housed under uniform environmental and management conditions. The room temperature was maintained at 23.8 °C, and the light period was 17 h/day. The flock was vaccinated (ND and IB) according to a routine schedule.

### 2.2. Production Performance Parameters

The formula for calculating feed intake (FI) was as follows: (FI = total feed offered − total feed used). Egg production was recorded on a daily basis. The formula for calculating hen-day egg production (HDEP) was as follows: (HDEP = total number of eggs in a particular time ÷ number of days × number of alive hens on each of these days). The weekly body weight gain (BWG) was calculated as (BWG = final body weight − starting body weight). Mortality and its causes were reported daily. Daily water intake was calculated by subtracting utilized water from given water. The formula used to calculate the feed conversion ratio (FCR) was (FCR = feed intake (kg) ÷ number of eggs × 12). [17].

### 2.3. Economics

The formula used to calculate total revenue (TR) was TR = total number of eggs × price per egg. The profit was found by subtracting the total cost from the total revenue. The cost–benefit ratio (CBR) was calculated by dividing the total revenue by the total cost. [18].

### 2.4. Egg Quality Traits

Four eggs were randomly selected from each replicate every week for four weeks to assess the internal and external quality. Individual eggs were weighed using a calibrated digital balance. The albumin and yolk were carefully separated from the eggshell, and the empty eggshell was dried overnight at 105 °C in a forced-air oven to ensure complete desiccation. Eggshell thickness, including the membranes, was measured at three locations on each egg using a micrometer screw gauge and averaged to provide a representative value. The egg contents were carefully transferred to a Petri dish, and albumin height was measured using a transparent plastic rod. The egg yolk was removed from the Petri dish using the suction technique, and the yolk weight was recorded. Finally, the separated albumin was weighed separately on the digital balance. Haugh units (HUs) were calculated using the equation outlined by [18,19]:

HU = 100log10 [H + 7.57 − 1.7W^0.37^] (1)

where H is the height of the albumen, and W is the weight of the egg.

### 2.5. Hematology and Serum Biochemistry

On the fourth week of the study, blood samples were collected from four birds in each replicate for the analysis of white blood cells (WBCs), red blood cells (RBCs), hemoglobin (Hb), and packed cell volume (PCV). Wright–Giemsa-stained blood smears were prepared and examined under a microscope to identify and count different types of WBCs. Manual counting using a hemocytometer was performed to determine the total number of WBCs and RBCs per unit of blood volume. The microhematocrit technique, using capillary tubes and centrifugation, was employed to measure the PCV occupied by RBCs. The cyanmethemoglobin method, based on the conversion of hemoglobin to cyanmethemoglobin and subsequent spectrophotometric measurement, was used to quantify the Hb concentration in blood. Serum lipid profiles were determined using a commercial kit developed by Cromatest^®^ Cholesterol MR (Linear Chemicals S.L., Barcelona, Spain). Total cholesterol (TC), high-density lipoprotein (HDL), and low-density lipoprotein (LDL) were analyzed in the blood serum. Very-low-density lipoprotein (VLDL) was calculated using the equation [20]

VLDL = TC − HDL − LDL(2)

### 2.6. Digesta Viscosity and Excreta Consistency

At the end of the experiment, three birds/replicate were euthanized by cervical dislocation. To measure the digesta viscosity, fresh digesta from the gizzard to Meckel’s diverticulum (proximal samples) and from Meckel’s diverticulum to the junction of the ileum, cecum, and colon (distal samples) were collected from two birds per replicate. Two samples were thoroughly mixed, and approximately 1.5 g of each sample was transferred to microcentrifuge tubes. After centrifuging the tubes for 5 min at 12,700× *g*, the supernatant was collected, and viscosity was measured using a digital viscometer (Brookfield, Brookfield, MA, USA) at a constant shear rate of 42.5 s^−1^ and a controlled temperature of 40 °C following established protocols [21]. The excreta consistency was visually assessed and scored in the fourth week of the experiment using a modified scoring system (Table 2) [20,22].

### 2.7. Apparent Total Tract Digestibility

The total collection method was used to determine nutrient digestibility. During the last three days of the experiment, three birds with similar average weights from each group were individually housed in metabolism cages equipped with separate feces collection trays. A known weight of feed was provided, and 1% Celite (Celite Corp., Lompoc, CA, USA) was added to all diets as an indigestible marker. Total excreta (including feces and urine) were collected twice daily (morning and evening) from each cage. Feathers and feed particles were carefully removed from the collected excreta, which were then weighed and stored at 4 °C for subsequent analysis. Feed and excreta samples were analyzed for dry matter (DM), crude protein (CP), crude fiber (CF), and ether extract (EE) following established protocols from AOAC (2000). The apparent digestibility of the nutrients was assessed using the index method according to the following equation, as outlined by [23,24]:

ATTD_X_, % = 100 − [(AIA_I_/AIA_O_) × (N_O_/N_I_) × 100] (3)

where ATTD_X_ is the apparent total tract digestibility of nutrients, AIA_I_ is the acid-insoluble ash concentration from dietary intake, AIA_O_ is the acid-insoluble ash concentration of fecal output, N_O_ is the nutrient concentration of fecal output, and N_I_ is the nutrient concentration of dietary intake.

### 2.8. Intestinal Histomorphology

Samples collected from the mid-regions of the duodenum, jejunum, and ileum were rinsed with 10× PBS (phosphate-buffered saline) and subsequently fixed in 10% buffered formalin for 24 h. After being preserved in 70% ethanol for 24 h, the samples were subjected to a progressive dehydration process that involved the use of increasing ethanol concentrations. This was followed by a clarifying process in xylene. The samples were immersed in paraffin wax and then cut into sections with a thickness of 5 μm. Two slices obtained at various depths were subjected to hematoxylin and eosin staining. Subsequently, images were captured at a magnification of 200× using a compound microscope (Nikon Eclipse 50, Nikon Corporation, Minato City, Tokyo, Japan). Five villi and crypts perpendicular to the muscularis mucosae and with a distinct border with the surrounding structure were chosen for further investigation. ImageJ software (version: 1.53u) was used to measure the width and height of five randomly chosen villi, as well as the depths of the crypts in each replication. The calculation of the villus surface area was performed using the formula provided by [20,25]:

Villus surface area = 2π × (Average villus width/2) × villus height(4)

### 2.9. Statistical Analysis

Using a Completely Randomized Design (CRD), the collected data were analyzed by the GLM procedure in SPSS 21.0 (IBM Corp, Armonk, NY, USA) using a one-way ANOVA model. Replicate pens were considered experimental units. Tukey’s test was applied to compare differences among the treatment groups. A *p*-value < 0.05 was considered statistically significant, and 0.05 ≤ *p*-value ≤ 0.1 indicated a trend toward statistical significance.

## 3. Results

### 3.1. Production Performance Parameters

The results of the effect of soyhulls and β-mannanase on the production performance of laying hens are shown in Table 3. Feed intake (FI), weight gain (WG), the feed conversion ratio (FCR), and water intake (WI) were significantly affected (*p* < 0.05) weekly as well as overall. FI during weeks 37, 39, and 40 had significantly higher (*p* < 0.05) values in the D2 group than in the remaining groups, while at week 38, FI was significantly higher (*p* < 0.05) in the D1, D2, and D4 groups compared to the control and D3 groups. Overall, a significantly higher (*p* < 0.05) FI was calculated in the D2 group as compared to the control and remaining combinations of soyhulls and β-mannanase. WG at weeks 37 and 40 was significantly higher (*p* < 0.05) in the D1, D2, and D4 groups compared to the control and D3 groups. At week 38, WG had a significantly higher (*p* < 0.05) value in the D1 and D2 groups than in the control, D3, and D4 groups. At week 39 and overall, WG was significantly higher (*p* < 0.05) in the D2 group as compared to the control and other combinations of soyhulls and β-mannanase. The FCR was significantly lower (better) (*p* < 0.05) in the D1 and D2 groups at weeks 37 and 39 but in the D1 group at week 38 than in the remaining groups. At week 40, the FCR was significantly lower (better) (*p* < 0.05) in the D2 and D4 groups as compared to the control and other combinations of soyhull and β-mannanase. Overall, a significantly lower (better) (*p* < 0.05) FCR was calculated in the D1 and D2 groups compared to the control and D3 and D4 groups. WI recorded at weeks 37, 38, 39, and 40 was significantly higher (*p* < 0.05) in the D1 and D2 groups than in the control, D3, and D4 groups. Overall, WI had a significantly higher (*p* < 0.05) value in the D2 group in comparison to the control and other soyhull and β-mannanase combinations. Egg production (EP), hen-day egg production (HDEP), and mortality were not different (*p* > 0.05) among the groups.

### 3.2. Economics

Table 4 indicates the effect of the soyhull and β-mannanase combinations on the economics of laying hens. Total revenue (TR) was calculated to be significantly higher (*p* < 0.05) in the D2 group compared to the control and other combinations of soyhulls and β-mannanase. Profit and cost–benefit ratio (CBR) was calculated significantly higher (*p* < 0.05) in the control than in the D2, D3, and D4 groups. Overall, economically, the D1 and D2 groups showed the best results as compared to the D3 and D4 groups.

### 3.3. Egg Quality Traits

Table 5 shows the effect of the soyhull and β-mannanase combinations on external egg quality (egg weight, shell weight, and shell thickness). No significant differences in external egg quality traits were observed among the control and treatment groups (*p* > 0.05). However, egg weights were numerically higher in the D2 group and shell weights in the D1 group compared to the control (no soyhulls, no enzyme) and other soyhull–enzyme combinations. Similarly, eggshell thickness exhibited numerically higher overall values (*p* > 0.05) in the control and D1 groups compared to the other groups. No significant differences (*p* > 0.05) were observed in egg yolk weight, albumen weight, albumen height, or Haugh unit among any groups throughout the study (Table 6). However, during the second week of the experiment (i.e., 38 weeks of age), albumen weight showed a significantly higher increase in the D2 group compared to the D3 group.

### 3.4. Hematology and Serum Biochemistry

Most hematological and serum biochemistry parameters were within normal ranges and showed no significant differences (*p* > 0.05) across the various soyhull–enzyme combinations (Table 7). These included red blood cell (RBC) count, white blood cell (WBC) count, heterophil percentage, lymphocyte percentage, packed cell volume (PCV), hemoglobin (Hb), mean corpuscular hemoglobin concentration (MCHC), mean corpuscular volume (MCV), high-density lipoprotein (HDL), heterophile/lymphocyte ratio (H:L), and total protein (TP). However, significant reductions were observed in total cholesterol (TC), low-density lipoprotein (LDL), and very-low-density lipoprotein (VLDL) levels for several soyhull–enzyme combinations compared to the control group. Notably, the D4 group displayed the lowest TC, LDL, and VLDL levels. Moreover, the D3 and D4 groups exhibited lower TC, LDL, and VLDL levels compared to the control, D1, and D2 groups.

### 3.5. Total Tract Digestibility, Digesta Viscosity, and Excreta Consistency

Table 8 summarizes the effects of different soyhull and β-mannanase combinations on total tract digestibility, digesta viscosity, and excreta consistency in laying hens. Dry matter (DM) digestibility was non-significant (*p* > 0.05) among the groups. Crude protein digestibility was significantly higher (*p* < 0.05) in the D2 group compared to the control and other combinations. Crude fiber, crude fat, and ash digestibility were significantly higher (*p* < 0.05) in the D1 and D2 groups compared to the D3, D4, and control groups. Digesta viscosity was numerically the highest in the D3 group, but no significant differences (*p* > 0.05) were found between the groups. Excreta consistency was similar in all groups, typical dry droppings with coning. In summary, the D1 and D2 groups seem the most beneficial for digestibility, particularly for dry matter, crude protein, fiber, fat, and ash. Higher soyhull or lower β-mannanase levels appear less effective in improving digestibility. Digesta viscosity, while not statistically significant, showed a trend toward higher values with higher soyhull and lower β-mannanase levels. All groups maintained normal excreta consistency, indicating no adverse effects on gut health.

### 3.6. Intestinal Histomorphology

The effects of different dietary combinations of soyhulls and β-mannanase on the villus morphology of the duodenum, jejunum, and ileum in laying hens are shown in Table 9. In the duodenum, the villus width (Vw) and height (Vh), crypt depth (Cd), and surface area (VSA) were significantly higher in the D2 group compared to the control group and other combinations. The villus surface area (VSA) was significantly higher in the D2 group compared to all other groups, further solidifying its improved absorptive capacity. In the jejunum, Vw was significantly higher in the D2 and D4 groups compared to the control and other combinations of soyhulls and β-mannanase. Vh was significantly higher in the D2 group than in the control, D1, D3, and D4 groups. Cd was significantly higher in the D1 and D2 groups compared to the control, D3, and D4 groups. The VSA followed the same pattern as Vh, with the D2 group showing the highest value and significant differences compared to the control, D3, and D4 groups. In the ileum, Vw followed a similar pattern to that in the jejunum, with the D2 and D4 groups having the highest values and significant differences compared to the control, D1, and D3 groups. Similar to the jejunum Vh, the ileum Vh was significantly higher in the D2 group compared to the control and other combinations. Cd was again significantly higher in the D1, D2, and D4 groups and lower in the control and D3 groups. The VSA followed the same pattern as Vh, with the D2 group showing the highest value and significant differences compared to the control and other combinations of soyhulls and β-mannanase. Overall, the D2 group appears to be the most beneficial for intestinal villus morphology in laying hens. This diet promotes increased villus height and width while maintaining the highest overall absorptive surface area.

## 4. Discussion

The late phase of peak egg production in laying hens (around 32–44 weeks of age) presents unique challenges. As hens age, their metabolic rate slows down, and their appetite naturally decreases [26]. Hens prioritize egg production, channeling a notable portion of their dietary intake toward yolk and albumen synthesis. This creates an increased demand for essential nutrients like protein, calcium, vitamins, and minerals, which further exacerbates the nutrient deficiency issue, creating a vicious cycle. Dietary fiber, often seen as an antagonist to productivity, can play a surprisingly valuable role in this crucial period. Including moderate fiber (2–4%) boosts beneficial gut bacteria, enhancing nutrient absorption, digestion, and immunity [27,28], while insoluble fiber can increase the feeding time and induce a feeling of fullness, potentially helping hens regulate their feed intake and preventing overconsumption [5,27]. Studies suggest that certain fiber sources (e.g., inulin and lignocellulose) might improve eggshell thickness and reduce the incidence of shell cracks and breaks [29,30]. The physical stimulation provided by some fiber sources might redirect foraging behavior and reduce feather pecking, a common welfare concern in laying hens [31]. Different fiber sources have varying digestibility and fermentability properties. Highly fermentable fibers, while beneficial for gut health, may reduce the energy available for egg production at high inclusion levels [32]. Recent research suggests that combining soyhulls with β-mannanase supplementation in their diets presents a promising avenue for achieving this balance [33,34,35]. Soyhulls, a readily available and cost-effective by-product of soybean processing, offer the potential to partially replace soybean meal in poultry diets, thereby reducing feed costs [36]. However, their high fiber content, primarily composed of non-starch polysaccharides like beta mannans, limits nutrient digestibility and might negatively impact egg production [15]. This is where B-mannanase, an enzyme specific to mannan degradation, plays a vital role. By breaking down these complex fibers, B-mannanase increases intestinal nutrient digestibility and utilization [37]. Several studies have reported its efficacy in laying hens, demonstrating improvements in egg production, the feed conversion ratio, and egg weight without compromising egg quality [38,39,40]. However, determining the optimal synergistic combination of soyhulls and B-mannanase is critical for realizing their full potential. While higher soyhull inclusion can further reduce feed costs, it might also necessitate a higher B-mannanase dosage to maintain efficient nutrient utilization.

Our study investigated the effects of adding different combinations of soyhulls and the β-mannanase enzyme to the diets of laying hens. The inclusion of 3% soyhulls and 30 mg/kg β-mannanase enzyme resulted in significantly higher overall feed intake, weight gain, and water intake and a better FCR than in the remaining groups, while egg production and mortality were not different among the groups. These results are in agreement with [41], who found that broilers fed 20% soya bean hull meal with 1% Safzyme had significantly higher feed intake and non-significant mortality. The inclusion of 100g/ton enzyme complex increased hen feed consumption compared to the control [42]. The current research findings align with those of [43], who observed increased feed intake in laying hens when enzymes were included in the feed at a concentration of 0.1-0.5 percent. The quantities of distiller’s dried grains with solubles (DDGS) and enzymes had a notable effect on feed efficiency (FCR) [44]. Similar to our findings, laying hens fed 10, 20, and 30% soybean husks with 2% cellulitic enzyme gained more weight than those fed them without the enzyme [45]. The results are consistent with [42], who found that 100 g/t of an enzyme complex (xylanase, ß-glucanase, and phytase-based) in feed formulations increased laying hens’ performance and egg production. Adding xylanase and phytase alone or in combination with wheat-based laying-hen diets with low phosphorus and corn–soya-based layer diets did not alter egg production [46,47,48]. Similar to the current investigation, feeding the treatment groups 0.1-0.5% enzyme increased the feed conversion ratio (from 2.15 to 2.03) [43]. Similarly, the provision of the enzyme Quatrazyme (20 mg/kg) in the feed of broilers improved the FCR from 2.11 to 1.99 [44]. The better feed intake in the soybean hull and enzyme diet groups is due to the beneficial effect of the enzyme on the gastrointestinal tract and its ability to break down the cell wall of the soybean hull into easily digestible components, and similarly, [49] determined that an increase in feed intake occurs solely after the viscosity is reduced by enzymatic supplementation, which degrades the NSP components of the diet. The increased body weight gain in the soybean hull and enzyme diet group is due to the improved feed intake. The better FCR in the 3% soyhull and 30 mg/kg β-mannanase enzyme diet group than in the remaining groups is a result of the comparatively high egg output in this group. The type of dietary fiber determines whether water intake rises or decreases, although ambient temperature, feed composition, and the physicochemical characteristics of diet constituents and components may influence this connection [50]. The findings of the current investigation are consistent with the significant correlation between feed intake and water intake [51], whereby the higher feed intake in the enzyme and SH groups led to a corresponding rise in water consumption.

The total revenue was higher (*p* < 0.05) in the 3% soyhull and 30 mg/kg β-mannanase enzyme diet group as compared to all other groups, while the profit and cost–benefit ratio (CBR) were higher (*p* < 0.05) in the control and 3% soyhull and 20 mg/kg β-mannanase groups than in the remaining groups, and similarly, negative feed cost savings and higher feed costs were calculated when using 10 and 20% soybean hull meal and 0.1% cellulitic enzyme (Safzyme) in the broiler finisher diet [41]. Similarly, adding alternative fiber sources (coffee husks and soybean hulls) and 0.075 g/kg xylanase to the diet of laying hens resulted in increased feed costs per egg carton [52]. Numerous factors, such as the type and quantity of cereal consumed, the amount of anti-nutritive ingredients in a specific cereal, the amounts of enzymes used, the animal’s age and type, the physiology of the bird, and the type of gut microflora, all affect the inclusion of enzymes in the diet and the improvement they achieve [53]. Due to increased egg production, the 3% soyhull and 30 mg/kg β-mannanase enzyme group had better overall income than the other groups, and the lower feed intake in the control group led to greater profits than in the other treatment groups. Feed, birds, eggs, and enzymes are all subject to market fluctuations, which primarily affect how dietary soybean hulls and enzymes are used in layer feed.

While statistically insignificant, we observed numerically higher egg weight in hens receiving 3% soyhulls and 30mg/kg enzyme. This finding aligns with previous research that also reported increased egg weight with enzyme supplementation in the diet [54,55,56]. However, other egg quality parameters, like the eggshell weight and thickness, yolk and albumen weight, albumen height, and Haugh unit, remained unaffected by our treatment. This suggests that while the enzyme may have slightly increased nutrient utilization and availability, it was not sufficient to significantly alter the proportions of different components within the egg.

Our study revealed a significant reduction in total cholesterol, LDL, and VLDL values with the combination of 9% soyhulls and 20 and 30 mg/kg enzyme compared to the control. These findings align with previous studies with the inclusion of higher levels of dietary fiber and a reduction in serum cholesterol levels in poultry [57,58,59]. Soyhulls contain both soluble and insoluble fiber portions, and the soluble portion of fiber lowers cholesterol by binding to it in the small intestine and preventing it from entering the bloodstream and exiting the body through the excreta [60,61]. The hematology and serum biochemistry parameters measured in our study all fell within the normal ranges [62]. This indicates that the dietary treatments did not negatively impact the overall health or internal physiology of the laying hens. Our findings align with previous studies reporting that the inclusion of up to 9% dietary fiber did not induce any significant changes in the internal physiology of the poultry [63,64]. This observation suggests that moderate levels of soyhulls with enzymes, as implemented in this study, are well tolerated and do not adversely affect the internal health of laying hens.

Our study indicated significantly higher total tract digestibility of crude protein, crude fiber, crude fat, and ash in the groups receiving 3% soyhulls and 20 or 30 mg/kg β-mannanase. This finding aligns with previous studies reporting improved digestibility with β-mannanase supplementation in poultry [65,66]. This may be explained by the fact that β-mannanase can hydrolyze the β-mannans in soyhulls, which are known to reduce nutrient digestibility by increasing the viscosity of intestinal digesta and inhibiting the activity of digestive enzymes [66,67]. By breaking down β-mannans, β-mannanase may reduce the viscosity of digesta, improve the mixing of enzymes and substrates, and increase the absorption of nutrients. The optimal level of β-mannanase for digestibility may depend on the content of β-mannans in the diet, as higher levels of β-mannans may require higher levels of β-mannanase to be effectively degraded. The results also indicate that higher levels of soyhulls or lower levels of β-mannanase may have negative effects on digestibility, as they may increase the viscosity of digesta and reduce the availability of nutrients. This is consistent with previous studies that reported the lower digestibility of dry matter, crude protein, fiber, and fat in pigs fed diets containing high levels of soyhulls or low levels of β-mannanase [67]. The increased viscosity of digesta may also affect intestinal morphology and health, as it may impair the mucosal barrier function and increase the susceptibility to pathogens [68]. However, in this study, no significant differences were observed in digesta viscosity or excreta consistency among the groups, suggesting that the levels of soyhulls and β-mannanase used in this study did not cause any adverse effects on gut health.

Our findings reveal a synergistic interaction between the combination of soyhulls and β-mannanase, significantly enhancing the intestinal villus structure and potentially improving nutrient absorption. In all three intestinal segments, hens fed the 3% soyhull and 30mg/kg β-mannanase diet exhibited the highest villus width and height. This observation aligns with previous studies demonstrating similar positive effects of moderate fiber inclusion and enzyme supplementation on gut morphology in poultry [68,69]. β-Mannanase specifically targets and degrades non-starch polysaccharides (NSPs) present in soyhulls, releasing trapped nutrients and potentially stimulating intestinal epithelial cell proliferation [70].

## 5. Conclusions

The findings of this study suggest that the combination of 3% soyhulls and 30mg/kg β-mannanase may offer a promising approach to improving the production performance, digestibility, gut health, cholesterol levels, and, potentially, economics of laying hens while maintaining acceptable egg quality.

## Figures and Tables

**Table 1:** Ingredient composition of experimental diets.

Nutrient %	Experimental Diets				
	CON	D1	D2	D3	D4
Soyhulls (%)	0	3	3	9	9
β-Mannanase (mg/kg)	0	20	30	20	30
Corn	53.1	52.1	52.1	50.5	50.5
Canola meal (34%)	4.15	3.85	3.67	2.16	2.14
Soybean meal (44%)	24.3	23.6	23.6	22.2	22.2
Guar meal	0	1	1	1	1
PBM Hi fat	2.00	1.02	1.00	1.02	0.85
Poultry Oil	2.79	2.79	2.72	2.67	2.67
Common Salt	0.32	0.32	0.41	0.26	0.41
Sodium bicarbonate	0.10	0.10	0.10	0.10	0.10
Limestone	11.1	10.1	10.3	8.98	9.157
Celite	1	1	1	1	1
Dicalcium phosphate	0.77	0.77	0.75	0.77	0.62
DL-Methionine	0.08	0.08	0.08	0.07	0.08
Choline Chloride (70%)	0.10	0.10	0.10	0.10	0.10
Vitamin Premix *	0.07	0.07	0.07	0.07	0.07
Mineral Premix *	0.06	0.06	0.06	0.06	0.06
Phytase	0.01	0.01	0.01	0.01	0.01
Enramycin	0.02	0.02	0.02	0.02	0.02
Ethoxyquin	0.01	0.01	0.01	0.01	0.01
NSPs	0.02	0.00	0.00	0.00	0.00
Total	100	100	100	100	100
Analyzed value					
Dry matter	89.4	90.5	90.6	91.0	91.1
Crude protein	17.7	17.5	17.5	17.0	17.0
Crude fiber	2.85	2.87	2.87	2.90	2.90
Crude fat	4.82	4.76	4.77	4.72	4.72
Ash	13.5	13.7	13.7	13.3	13.4
Moisture	10.5	9.48	9.38	9.00	8.84
Nitrogen-Free Extract	51.6	49.0	49.0	45.8	45.8

* To provide 1 kg of diet: Retinyl acetate, 4400 IU; DL-α-tocopheryl acetate, 12 IU; Cholecalciferol, 118 µg; Thiamine, 2.5 mg; Menadione sodium bisulfite, 2.40 mg; Niacin, 30 mg; Vit. B_2_, 4.8 mg; D-pantothenic acid, 10 mg; Vit. B_6_, 5 mg; Vit. B_7_, 130 µg; Cyanocobalamine, 19 µg; Vit. B_9_, 2.5 mg; Mn, 85 mg; Zinc, 75 mg; Fe, 80 mg; Iodine, 1 mg; Selenium, 130 µg; Copper, 6 mg. PBM, Poultry by-product meal; DCP, Dicalcium phosphate; DLM, DL-Methionine; NSPs, non-starch polysaccharides. CON, control; D1 = 3%SH + 20 mg/kg β-mannanase; D2 = 3%SH + 30 mg/kg; D3 = 9%SH + 20 mg/kg; D4 = 9%SH + 30 mg/kg.

**Table 2:** Scoring of excreta consistency.

Score	Description
1	Typical dry droppings with coning
2	No free water, loose droppings, and some coning.
3	Loose droppings, minor coning, and some free water
4	Very loose, non-coning droppings and copious quantities of free water

**Table 3:** Effects of the dietary inclusion of different combinations of soyhulls and β-mannanase on the production performance of the laying hens.

Diets ^1^	Weeks	CON	D1	D2	D3	D4	SEM	*p*-Value
Soyhulls (%)	-----	0	3	3	9	9		
β-Mannanase (mg/kg)	-----	0	20	30	20	30		
Feed intake (g)	37	770 ^c^	778 ^b^	787 ^a^	770 ^c^	778 ^b^	0.88	0.047
	38	783 ^c^	798 ^a^	801 ^a^	794 ^b^	797 ^a^	1.06	0.042
	39	765 ^d^	786 ^b^	793 ^a^	780 ^c^	788 ^b^	1.12	0.031
	40	790 ^c^	804 ^b^	813 ^a^	792 ^c^	806 ^b^	0.96	0.011
	Overall	3108 ^d^	3166 ^b^	3194 ^a^	3136 ^c^	3169 ^b^	7.02	0.021
Weight gain (g)	37	13.0 ^b^	14.0 ^ab^	17.2 ^a^	13.0 ^b^	14.7 ^ab^	1.04	0.019
	38	22.0 ^b^	23.0 ^ab^	24.5 ^a^	18.0 ^c^	21.0 ^bc^	1.41	0.034
	39	20.0 ^b^	18.0 ^c^	23.0 ^a^	21.0 ^b^	21.2 ^b^	1.48	0.046
	40	12.0 ^c^	14.0 ^ab^	15.2 ^a^	13.0 ^bc^	14.0 ^ab^	1.29	0.041
	Overall	65.4 ^c^	69.0 ^b^	80.0 ^a^	64.2 ^c^	71.0 ^b^	3.42	0.039
Egg production/bird	37	5.00	5.15	5.18	5.00	5.04	0.82	0.720
	38	5.00	5.20	5.17	5.00	5.04	0.76	0.611
	39	4.88	5.06	5.08	4.93	5.02	0.92	0.454
	40	4.87	4.98	5.10	4.89	5.02	0.58	0.150
	Overall	19.7	20.3	20.5	19.8	20.1	0.30	0.062
FCR	37	1.84 ^a^	1.81 ^b^	1.82 ^b^	1.84 ^a^	1.85 ^a^	0.03	0.033
	38	1.87 ^b^	1.84 ^c^	1.86 ^b^	1.90 ^a^	1.89 ^a^	0.08	0.015
	39	1.88 ^a^	1.86 ^b^	1.87 ^ab^	1.89 ^a^	1.88 ^a^	0.06	0.046
	40	1.94 ^a^	1.93 ^ab^	1.91 ^c^	1.94 ^a^	1.92 ^bc^	0.12	0.038
	Overall	1.88 ^a^	1.86 ^b^	1.86 ^b^	1.89 ^a^	1.89 ^a^	0.14	0.049
Water intake (liter/bird)	37	1.23 ^c^	1.27 ^ab^	1.28 ^a^	1.24 ^c^	1.26 ^b^	0.34	0.001
	38	1.24 ^c^	1.27 ^ab^	1.28 ^a^	1.26 ^b^	1.26 ^b^	0.93	0.001
	39	1.26 ^c^	1.29 ^ab^	1.31 ^a^	1.28 ^b^	1.28 ^b^	0.61	0.006
	40	1.25 ^c^	1.28 ^ab^	1.29 ^a^	1.25 ^c^	1.27 ^b^	2.31	0.012
	Overall	5.02 ^c^	5.12 ^b^	5.18 ^a^	5.05 ^c^	5.07 ^bc^	0.49	0.002
HDEP %	37	71.4	73.4	74.0	71.4	72.0	1.11	0.143
	38	71.4	74.2	74.0	71.4	72.0	1.19	0.239
	39	70.4	72.2	72.4	70.6	72.0	1.14	0.146
	40	70.2	71.4	73.0	70.5	72.0	1.10	0.117
	Overall	71.0	73.0	73.3	71.4	72.0	0.39	0.212
Mortality %	37	0.00	0.00	0.00	0.00	0.00	----	----
	38	0.00	0.00	0.00	0.00	0.00	----	----
	39	0.00	0.00	0.00	0.00	0.00	----	----
	40	0.00	0.00	0.00	0.00	0.00	----	----
	Overall	0.00	0.00	0.00	0.00	0.00	----	----

The means within a row that have different superscripts show a significant difference (*p* < 0.05). ^1^ CON, control; D1 = 3%SH + 20 mg/kg β-mannanase; D2 = 3%SH + 30 mg/kg; D3 = 9%SH + 20 mg/kg; D4 = 9%SH + 30 mg/kg. FCR = feed conversion ratio; HDEP = hen-day egg production.

**Table 4:** Effects of the dietary inclusion of different combinations of soyhulls and β-mannanase on the economics of laying hens.

Diets ^1^	CON	D1	D2	D3	D4	SEM	*p*-Value
Soyhulls (%)	0	3	3	9	9		
β-Mannanase (mg/kg)	0	20	30	20	30		
Total revenue (R.s) ^2^	324 ^d^	334 ^b^	337 ^a^	326 ^d^	331 ^c^	1.18	0.002
Profit (R.s) ^3^	132 ^a^	130 ^ab^	127 ^b^	124 ^c^	125 ^bc^	1.44	0.016
Cost–benefit ratio	1.59 ^a^	1.57 ^ab^	1.55 ^b^	1.52 ^c^	1.51 ^c^	0.03	0.002

The means within a row that have different superscripts show a significant difference (*p* < 0.05). ^1^ CON = control; D1 = 3%SH + 20 mg/kg β-mannanase; D2 = 3%SH + 30 mg/kg; D3 = 9%SH + 20 mg/kg; D4 = 9%SH + 30 mg/kg. ^2^ R.s = Rupees. ^3^ Profit was calculated as the difference between total revenue and total cost (feed, vaccination and medication, labor, electricity, and other miscellaneous costs).

**Table 5:** Effects of the dietary inclusion of different combinations of soyhulls and β-mannanase on the external egg quality.

Diets ^1^	Week	CON	D1	D2	D3	D4	SEM	*p*-Value
Soyhulls (%)	-----	0	3	3	9	9		
β-Mannanase (mg/kg)	-----	0	20	30	20	30		
Egg weight (g)	37	56.5	57.0	57.7	56.8	57.3	0.64	0.706
	38	56.7	57.1	57.6	56.8	57.5	0.75	0.738
	39	57.0	57.6	58.0	57.4	58.2	0.78	0.765
	40	57.2	57.8	58.6	57.8	58.0	1.08	0.852
	Overall	56.8	57.4	58.0	57.2	57.7	0.57	0.441
Shell weight (g)	37	5.72	6.03	6.16	5.89	6.08	0.22	0.298
	38	5.22	5.73	5.81	5.39	5.58	0.18	0.100
	39	5.33	5.63	5.74	5.44	5.53	0.26	0.440
	40	5.54 ^b^	5.73 ^a^	5.90 ^a^	5.57 ^b^	5.64 ^ab^	0.14	0.004
	Overall	5.50	5.82	5.74	5.50	5.70	0.09	0.220
Shell thickness (mm)	37	0.32	0.33	0.31	0.32	0.33	0.05	0.933
	38	0.35	0.33	0.34	0.33	0.31	0.20	0.368
	39	0.35	0.36	0.34	0.34	0.35	0.07	0.655
	40	0.34	0.34	0.34	0.33	0.35	0.05	0.451
	Overall	0.34	0.34	0.33	0.33	0.33	0.04	0.477

The means within a row that have different superscripts show a significant difference (*p* < 0.05). ^1^ CON = control; D1 = 3%SH + 20 mg/kg β-mannanase; D2 = 3%SH + 30 mg/kg; D3 = 9%SH + 20 mg/kg; D4 = 9%SH + 30 mg/kg.

**Table 6:** Effects of the dietary inclusion of different combinations of soyhulls and β-mannanase on the internal egg quality.

Diets ^1^	Week	CON	D1	D2	D3	D4	SEM	*p*-Value
Soyhulls (%)	-----	0	3	3	9	9		
β-Mannanase (mg/kg)	-----	0	20	30	20	30		
Yolk weight (g)	37	17.6	17.0	17.2	18.2	17.0	0.58	0.776
	38	17.0	16.6	16.5	17.7	16.7	0.82	0.873
	39	17.3	16.8	17.4	17.4	16.6	0.64	0.897
	40	17.0	17.2	17.6	18.1	17.2	0.49	0.675
	Overall	17.2	16.9	17.2	17.8	16.9	0.70	0.803
Albumen weight (g)	37	32.7	33.1	33.6	31.97	33.5	0.46	0.186
	38	33.9 ^ab^	34.6 ^a^	35.0 ^a^	32.8 ^b^	34.7 ^a^	0.39	0.004
	39	34.1	34.9	34.6	34.0	35.0	0.60	0.710
	40	34.2	33.9	35.0	33.8	34.8	0.51	0.293
	Overall	33.7	34.1	34.5	33.5	34.4	0.27	0.061
Albumen height (mm)	37	6.73	7.00	7.08	6.88	6.95	0.24	0.881
	38	6.83	7.10	7.18	6.98	7.05	0.19	0.884
	39	6.65	6.90	7.08	6.75	6.80	0.22	0.660
	40	6.35	6.60	6.78	6.45	6.50	0.28	0.663
	Overall	6.64	6.90	7.03	6.76	6.83	0.17	0.689
Haugh unit	37	76.5	77.5	78.4	75.6	76.7	1.09	0.706
	38	76.7	77.7	78.4	77.4	78.6	1.84	0.946
	39	75.4	76.6	76.9	75.5	76.0	1.84	0.945
	40	76.2	76.9	77.2	76.4	77.0	1.21	0.991
	Overall	76.2	77.2	77.7	76.2	77.0	0.74	0.338

The means within a row that have different superscripts show a significant difference (*p* < 0.05). ^1^ CON = control; D1 = 3%SH + 20 mg/kg β-mannanase; D2 = 3%SH + 30 mg/kg; D3 = 9%SH + 20 mg/kg; D4 = 9%SH + 30 mg/kg.

**Table 7:** Effects of the dietary inclusion of different combinations of soyhulls and β-mannanase on hematological and serum biochemical indices in laying hens at 40 weeks of age.

Diets ^1^	CON	D1	D2	D3	D4	SEM	*p*-Value
Soyhulls (%)	0	3	3	9	9		
β-Mannanase (mg/kg)	0	20	30	20	30		
Hematological Indices ^2^							
RBCs (10^6^μL)	3.17	3.21	3.23	3.18	3.20	0.02	0.070
WBCs (10^3^μL)	3.10	3.06	3.05	3.07	3.08	0.01	0.061
Heterophile (%)	22.0	23.0	24.0	22.0	23.1	1.50	0.675
Lymphocyte (%)	58.0	59.1	59.2	58.2	58.7	1.19	0.765
H:L	0.38	0.38	0.40	0.37	0.39	0.03	0.857
PCV (%)	27.4	30.0	30.1	27.8	28.9	0.64	0.063
HB (g/dL)	10.3	11.0	11.2	10.4	10.5	0.12	0.061
MCHC (g/dL)	38.0	37.0	37.2	37.4	36.3	0.42	0.062
MCV (fL)	88.8	91.4	91.1	89.4	90.3	1.95	0.074
Serum Biochemical Indices ^3^							
TC (mg/dL)	132 ^a^	125 ^b^	124 ^b^	122 ^c^	121 ^c^	1.62	0.007
HDL (mg/dL)	67.0	67.0	68.0	70.0	70.0	1.41	0.129
LDL (mg/dL)	39.2 ^a^	37.0 ^ab^	35.3 ^b^	33.4 ^c^	32.1 ^c^	1.06	0.002
VLDL (mg/dL)	25.4 ^a^	21.0 ^b^	21.3 ^b^	18.3 ^c^	18.0 ^c^	2.02	0.042
TP (mg/dL)	5.00	5.07	5.12	5.04	5.10	0.02	0.074

The means within a row that have different superscripts show a significant difference (*p* < 0.05). ^1^ CON, control; D1 = 3%SH + 20 mg/kg β-mannanase; D2 = 3%SH + 30 mg/kg; D3 = 9%SH + 20 mg/kg; D4 = 9%SH + 30 mg/kg. ^2^ Red blood cells (RBCs); white blood cells (WBCs); heterophil-to-lymphocyte ratio (H:L); packed cell volume (PCV); hemoglobin (Hb); mean corpuscular hemoglobin concentrations (MCHC); mean corpuscular volume (MCV). ^3^ Total cholesterol (TC); high-density lipoprotein (HDL); low-density lipoprotein (LDL); very-low-density lipoprotein (VLDL); total protein (TP).

**Table 8:** Effects of the dietary inclusion of soybean hulls and enzyme on the total tract digestibility, digesta viscosity, and excreta consistency.

Diets ^1^	CON	D1	D2	D3	D4	SEM	*p*-Value
Soyhulls (%)	0	3	3	9	9		
β-Mannanase (mg/kg)	0	20	30	20	30		
Dry matter %	79.0	81.4	82.0	80.0	82.0	1.02	0.062
Crude protein %	68.0 ^c^	71.0 ^b^	74.0 ^a^	68.0 ^c^	70.0 ^b^	1.38	0.029
Crude fiber %	68.0 ^c^	71.0 ^ab^	72.0 ^a^	69.0 ^bc^	69.0 ^bc^	1.03	0.005
Crude fat %	75.0 ^c^	80.0 ^a^	80.4 ^a^	77.0 ^b^	77.4 ^b^	0.88	0.003
Ash %	56.2 ^c^	59.0 ^ab^	60.2 ^a^	58.0 ^b^	58.0 ^b^	0.87	0.007
Viscosity (cP)	4.76	4.73	4.70	5.02	4.78	0.16	0.521
Excreta consistency ^2^	1	1	1	1	1		------

The means within a row that have different superscripts show a significant difference (*p* < 0.05). ^1^ CON, control; D1 = 3%SH + 20 mg/kg β-mannanase; D2 = 3%SH + 30 mg/kg; D3 = 9%SH + 20 mg/kg; D4 = 9%SH + 30 mg/kg. ^2^ For excreta consistency, ‘1’ shows normal dry droppings and cone formation. Centipoise, cP.

**Table 9:** Effects of dietary inclusion of soyhulls and enzyme (β-mannanase) on intestinal histomorphology.

Diets ^1^	CON	D1	D2	D3	D4	SEM	*p*-Value
Soyhulls (%)	0	3	3	9	9		
β-Mannanase (mg/kg)	0	20	30	20	30		
Duodenum ^2^							
Vw (um)	75.0 ^d^	83.0 ^b^	89.0 ^a^	79.0 ^c^	83.0 ^b^	2.65	0.022
Vh (um)	595 ^d^	613 ^b^	621 ^a^	604 ^c^	611 ^b^	6.64	0.028
Cd (um)	100 ^d^	107 ^b^	111 ^a^	102 ^cd^	103 ^d^	4.10	0.036
VSA (mm^2^)	0.139 ^d^	0.160 ^b^	0.172 ^a^	0.149 ^c^	0.158 ^b^	0.04	0.006
Jejunum							
Vw (um)	69.0 ^c^	72.0 ^b^	76.0 ^a^	70.0 ^c^	74.0 ^ab^	2.39	0.039
Vh (um)	447 ^d^	459 ^c^	470 ^a^	457 ^c^	466 ^b^	5.44	0.021
Cd (um)	53.0 ^d^	69.0 ^ab^	71.0 ^a^	61.0 ^c^	67.0 ^b^	2.37	0.009
VSA (mm^2^)	0.096 ^d^	0.103 ^b^	0.111 ^a^	0.100 ^b^	0.107 ^c^	0.04	0.007
Ileum							
Vw (um)	62.0 ^c^	65.0 ^b^	68 ^a^	63.0 ^bc^	67.0 ^a^	2.19	0.043
Vh (um)	397 ^d^	407 ^b^	412 ^a^	403 ^c^	401 ^c^	4.88	0.032
Cd (um)	59.0 ^bc^	60 ^ab^	62.0 ^a^	58.0 ^c^	60.0 ^ab^	2.12	0.032
VSA (mm^2^)	0.078 ^c^	0.083 ^b^	0.086 ^a^	0.079 ^c^	0.082 ^b^	0.03	0.038

The means within a row that have different superscripts show a significant difference (*p* < 0.05). ^1^ CON, control; D1 = 3%SH + 20 mg/kg β-mannanase; D2 = 3%SH + 30 mg/kg; D3 = 9%SH + 20 mg/kg; D4 = 9%SH + 30 mg/kg. ^2^ Vw = villus width; Vh = villus height; Cd = crypt depth; Vh: Cd = villus height to crypt depth ratio; VSA = villus surface area.

## Data Availability

The data that support the findings of this study are available from the corresponding author upon reasonable request.
